# Application of β-Cyclodextrin Adsorbents in the Removal of Mixed Per- and Polyfluoroalkyl Substances

**DOI:** 10.3390/toxics12040264

**Published:** 2024-03-31

**Authors:** Elham Abaie, Manish Kumar, Naveen Kumar, Yilang Sun, Jennifer Guelfo, Yuexiao Shen, Danny Reible

**Affiliations:** 1 Civil, Environmental, and Construction Engineering Department, Texas Tech University, Lubbock, TX 79409, USA; elham.abaie@ttu.edu (E.A.); maniskum@ttu.edu (M.K.); naveengupta266@gmail.com (N.K.); yilang.sun@ttu.edu (Y.S.); jennifer.guelfo@ttu.edu (J.G.); 2 Chemical Engineering Department, Texas Tech University, Lubbock, TX 79409, USA

**Keywords:** PFAS, groundwater contamination, β-cyclodextrin, adsorption, fluorine-free adsorbents

## Abstract

The extensive use of per- and polyfluoroalkyl substances (PFASs) in industrial consumer products has led to groundwater contamination, raising concerns for human health and the environment. These persistent chemicals exist in different forms with varying properties, which makes their removal challenging. In this study, we assessed the effectiveness of three different β-cyclodextrin (β-CD) adsorbents at removing a mixture of PFASs, including anionic, neutral, and zwitterionic compounds, at neutral pH. We calculated linear partition coefficient (K_d_) values to quantify the adsorption affinity of each PFAS. β-CD polymers crosslinked with hexamethylene diisocyanate (β-CD-HDI) and epichlorohydrin (β-CD-EPI) displayed some adsorption of PFASs. Benzyl chloride β-CD (β-CD-Cl), an adsorbent that had not been previously reported, was also synthesized and tested for PFAS adsorption. β-CD-Cl exhibited higher PFAS adsorption than β-CD-HDI and β-CD-EPI, with log K_d_ values ranging from 1.9 L·g^−1^ to 3.3 L·g^−1^. β-CD-Cl displayed no affinity for zwitterionic compounds, as opposed to β-CD-HDI and β-CD-EPI, which removed N-dimethyl ammonio propyl perfluorohexane sulfonamide (AmPr-FHxSA). A comparison between K_d_ values and the log K_ow_ of PFAS confirmed the significant role of hydrophobic interactions in thee adsorption mechanism. This effect was stronger in β-CD-Cl, compared to β-CD-HDI and β-CD-EPI. While no effect of PFAS charge was observed in β-CD-Cl, some influence of charge was observed in β-CD-HDI and β-CD-EPI, with less negative compounds being more adsorbed. The adsorption of PFASs by β-CD-Cl was similar in magnitude to that of other adsorbents proposed in literature. However, it offers the advantage of not containing fluorine, unlike many commonly proposed adsorbents.

## 1. Introduction

The occurrence of per- and polyfluoroalkyl substances (PFASs) in the environment has raised significant concern, due to the potential harm they can pose on both human health and the ecosystem. The widespread use and persistence of PFASs have led to their accumulation in various environmental compartments, including soil, water bodies, and even the atmosphere. Of particular concern is their contamination of drinking water sources, which has raised alarming questions about the potential long-term consequences for both the environment and human health [1]. Water resources can contain various types of PFASs, including well-known compounds such as perfluorooctanoic acid (PFOA) and perfluorooctanesulfonic acid (PFOS), as well as emerging PFASs. 

To date, there have been numerous efforts to apply various treatment technologies aimed at eliminating PFASs. Notably, the adsorption of PFASs onto different matrices has received extensive research attention. Among these techniques, granular activated carbon (GAC) and ion-exchange resins (Ixs) have emerged as well-established sorption methods, showing promise in effectively removing PFOS and PFOA. Due to their favorable attributes, including cost-effectiveness, operational simplicity, and high removal efficiency, GAC and Ixs remain preferred options for PFAS remediation. However, destructive methods such as chemical oxidation, chemical reduction, electrochemical, and sonochemical approaches have not yet proven feasible in achieving the complete degradation of PFASs [2,3].

GAC and Ixs have gained popularity as effective methods for PFAS treatment; however, their application has been associated with several challenges. GAC has shown excellent removal rates for long-chain PFASs, but its performance diminishes when dealing with shorter-chain PFASs [3]. On the other hand, ion-exchange resins have exhibited enhanced capability in removing shorter-chain PFASs. Nevertheless, the presence of additional organic contaminants can have an adverse effect on the removal efficiency of both GAC and Ixs due to fouling. Furthermore, both GAC and Ixs are plagued by limited regenerability. This necessitates frequent replacements, resulting in elevated operational costs [1]. 

While most investigations into PFAS removal using GAC or Ixs have primarily focused on PFOA and PFOS remediation, the complex identity of various PFASs present in contaminated groundwater challenges the applicability of results attained from previous investigations on a limited number of PFASs [4,5]. Limited research into the removal of multiple PFASs using GAC, as well as combinations of GAC with resin or membrane, has indicated greater sorption rates for longer-chain and linear PFASs, whereas reduced removal efficiencies and earlier breakthrough have been noted for shorter-chain and branched PFASs [6,7,8,9]. These results align with simulation works on a mixture of PFASs [10,11]. Meanwhile, the co-removal of PFASs using destructive methods results in an incomplete destruction of PFASs, short-chain PFAS by-products, and the release of undesirable chemicals [12,13,14,15]. Shorter-chain PFASs exhibit increased mobility, greater resistance to breakdown, and a higher potential for bioaccumulation in organisms. Moreover, the incomplete destruction of PFAS compounds could lead to the generation of unknown by-products, posing potential environmental hazards and increased difficulty in management [16]. 

Cyclodextrin-based adsorbents have gained attention in recent years for their effectiveness in the removal of PFASs from water and other environmental sources. Cyclodextrin (CD) molecules have a distinctive torus shape and Angstrom-scale cavity diameter, making them excellent building blocks for adsorption materials. Furthermore, CDs contain numerous -OH groups that allow them to be modified or functionalized for use in various adsorption applications [15].

Considerable efforts have been dedicated to the synthesis and evaluation of various β-CD polymers for PFAS removal. While the conventional β-CD polymer, such as β-CD crosslinked with epichlorohydrin (EPI), exhibits only limited affinity for PFASs, recent research has shifted its focus toward alternative crosslinkers with aromatic groups, such as tetrafluoroterephthalonitrile (TFN), which incorporates multiple fluorine groups in its structure [17,18,19,20,21,22,23,24,25,26]. Although these adsorbents have demonstrated rapid and efficient adsorption of certain PFASs, the addition of fluorine groups may introduce additional environmental concerns related to their use. Therefore, more attention has been given to non-fluorinated adsorbents for PFAS removal. In recent studies, fluorine-free styrenic β-CD polymers were synthesized and tested successfully for anionic PFAS removal [27,28]. Despite the effectiveness of these polymers in PFAS removal, their synthesis process is intricate and involves multiple steps.

In the current work, three different types of β-CD adsorbents were synthesized, including two conventional β-CD polymers: β-CD crosslinked with EPI (β-CD-EPI), β-CD crosslinked with hexamethylene diisocyanate (β-CD-HDI), and a novel sorbent, chlorine-functionalized β-CD (β-CD-Cl). Previous studies suggested that crosslinker chemistry plays a key role in the adsorption mechanism of PFASs [17]. It is also suggested that protonation of amine functional groups gives a positive charge to the adsorbent, which would make the adsorbent more favorable for anionic PFASs. Electrostatic interactions between the negatively charged carboxylate or sulfonate headgroup of the anionic PFAS and the positively charged amines of the adsorbent were claimed to play a predominant role in the adsorption of PFASs onto amine-functionalized β-CD adsorbents [25]. To investigate the impact of amine functional groups on PFAS uptake, β-CD-HDI was synthesized. It was hypothesized that the protonation of amine groups in isocyanate gives positive charge to the adsorbent surface and makes it more favorable for the adsorption of anionic PFASs. On the other side, β-CD-EPI was selected as a macrocyclic adsorbent that lacks significant ionizable groups, allowing for a comparison with β-CD-HDI. 

In addition to polymerized β-CD adsorbents, β-CD-Cl was also synthesized as a functionalized β-CD adsorbent to investigate the role of polar groups in the removal of PFASs. In the current study, β-CD-Cl was recommended as a fluorine-free adsorbent, similar to styrenic β-CD polymers [27] but with an easier synthesis process. In this study, chlorine was selected as a highly electronegative atom to be incorporated into the β-CD adsorbents. The hypothesis was that the addition of chlorine would enhance the polarity of this adsorbent, similar to fluorinated β-CD adsorbents, with the distinction that fluorine was not introduced into the adsorbent. 

While β-CD-Cl has not been tested for PFAS removal, both β-CD-HDI and β-CD-EPI have been previously explored for PFAS removal. In a prior study, an adsorption capacity that ranged from 1.3 mmol·g^−1^ to 2.6 mmol·g^−1^ of PFOA was reported for β-CD-HDI [29]. In another study, approximately 15–20% removal of PFOA was observed at C_0_ = 1 μg·L^−1^ and at an adsorbent dosage of 10 mg·L^−1^. In the current work, the adsorption affinities of all three adsorbents for different PFASs in a mixed media were measured. The performances of these adsorbents were also compared to four commercially available adsorbents that were studied for PFAS removal. 

## 2. Materials and Methods

### 2.1. Chemicals and Reagents

Reagents were purchased from Millipore-Sigma (Burlington, MA, USA) and used as received. All the adsorbents were ground with a mortar and pestle to achieve a fine powder. The 15 mL VWR^®^ High-Performance Centrifuge Tubes(were used for adsorption experiments. PFAS stock solution was kindly provided by Dr. Jennifer Guelfo at Texas Tech University. The PFAS stock solution contained 27 different compounds: twenty-three anionic, one non-ionic, and three zwitterionic PFASs. Key characteristics of studied PFASs are provided in Table S1.

### 2.2. Design and Synthesis of Adsorbents

The unique structure of cyclodextrins allows their polymerization with crosslinking agents. Β-CD polymers were previously explored for PFOA removal, and a higher affinity of β-CD toward PFOA compared to GAC was observed [23]. In the current work, cyclodextrins were chosen to synthesize the adsorbent due to the promising results from the previous studies. 

Several adsorption mechanisms were reported for β-CD adsorbents. The adsorption primarily involves the formation of host–guest complexes through non-covalent interactions [30]. The host–guest interaction is a phenomenon where two molecules or ions form complexes through unique structural associations and non-covalent binding. A notable feature of this interaction is the presence of cavities within host molecules, which are often customized and fine-tuned in terms of their dimensions, configuration, and chemical characteristics to match specific targets [31]. 

Moreover, β-CD is characterized by a hydrophobic surface, which allows hydrophobic interactions to play a key role in adsorption. Several studies on the application of β-CD adsorbents in the removal of PFASs have proven the effect of hydrophobic interactions in adsorption [10,25,26,32]. Previous studies investigating the adsorption of PFASs on various adsorbent materials, including β-CD adsorbents, have indicated that electrostatic and hydrophobic interactions are the primary driving forces [32]. Hydrophobic interaction was found to be enhanced with increasing C–F chain length [31]. On the other hand, studies suggest that longer-chain PFAS compounds tend to be more efficiently removed through hydrophobic interactions, while shorter-chain ones are influenced more by electrostatic interactions [32]. Similar to hydrophobic interactions, fluorophobic interactions are also proposed for PFAS binding, as many studies showed that fluorinated probes had better adsorption performance than nonfluorinated ones [19,22,23]. 

Additional factors affecting PFAS adsorption include hydrogen bonding, covalent bonding, and the molecular structure of PFASs [31]. Hydrogen bonding could be formed between the polar headgroups of PFASs, with the hydrogen atoms bonded to either nitrogen or oxygen in the functional groups on material surfaces. However, competitive hydrogen bonding with material surfaces will happen between water molecules and PFAS molecules [31]. 

Previous studies on the role of the crosslinker in the affinity of β-CD polymers toward PFOA and PFOS indicated that polymers with cationic functional groups remove anionic PFASs effectively, while a lower affinity of the anionic β-CD polymer toward PFOA was reported [22]. In the current work, hexamethylene diisocyanate (HDI) and EPI were selected as crosslinkers to synthesize cationic and neutral β-CD polymers. The resulting β-CD-HDI and β-CD-EPI polymers were both obtained as insoluble white amorphous powders, which underwent vacuum drying. Subsequently, the polymers were ground using a mortar and pestle, and the resulting material was passed through a 40-mesh sieve to ensure a uniform particle size. Moreover, the functionalized β-CD polymer was synthesized by adding chlorine functional groups to β-CD, as described in the literature [33,34,35]. The synthesis process introduced benzyl chloride groups into β-CD, enhancing its water-insoluble properties and incorporating CH_2_Cl groups, which provide polar areas to the β-CD adsorbent. The resulting adsorbent was a yellow amorphous powder that underwent vacuum drying and was sieved using a 40-mesh sieve to achieve a uniform particle size.

**Figure 1:**
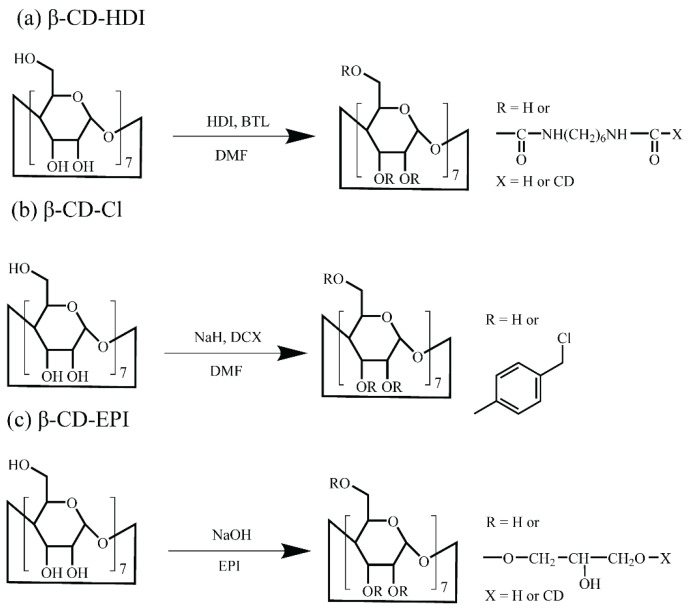
Synthesis paths of β-CD-HDI (**a**), β-CD-Cl (**b**), and β-CD-EPI (**c**) adsorbents.

Fourier transform infrared (FTIR) analysis was conducted to confirm that the desired polymer was attained (see Figures S1–S3). Figure 1 shows a scheme of the synthesis path for three adsorbents that were evaluated in this work. 

β-CD-HDI and β-CD-EPI adsorbents were previously characterized [36,37] and exhibited modest surface areas (1–5 m^2^·g^−1^), as measured by N_2_ adsorption BET. Characteristics of the β-CD-Cl were not previously reported, and BET sorption, SEM images, and XRD analysis were reported in the Supplementary Information. The β-CD-Cl are amorphous particles, 10–30 µm in diameter, with a surface area of 8.9 m^2^·g^−1^ and a dominant pore size of 20 nm (Figures S4–S6). The modest BET surface areas is similar to that of other β-CD materials, reflecting the relatively poor nitrogen adsorption of these materials [36]. Wilson et al. [36] showed much higher surface areas in aqueous solutions and attributed it to the swelling of the copolymer framework in aqueous solutions. Although the BET N_2_ adsorption may be misleading for this reason, it is a convenient and common tool for the comparison of the various sorbents, showing that β-CD-Cl exhibits a surface area approximately double the other β-CD polymers employed here. 

### 2.3. Adsorption Experiments

Adsorption experiments were designed based on our experience from working with these adsorbents for other compounds and information derived from literature [17,18,19,20,22,23,25,26]. Previous studies suggest that, given PFASs are present at very low concentrations in the environment, higher concentrations would not provide information about environmentally relevant conditions [17]. Therefore, adsorption capacities were evaluated at 100 ng·L^−1^ and 1000 ng·L^−1^ only. Adsorption experiments were conducted using three different adsorbents, as described, at an adsorbent dosage of 10 mg·L^−1^ in pH-neutral water. Adsorbents were added to 15 mL centrifuge tubes. The tubes were filled with 10 mL of nano-pure water. Then, an appropriate amount of PFAS stock solution was spiked in each tube to reach the desired concentration. Adsorption experiments were conducted at initial PFAS concentrations of 100 ng·L^−1^ and 1000 ng·L^−1^. Tubes were slowly mixed with a rotating mixer) for 5 h to ensure equilibration. Samples were centrifuged for 30 min to separate adsorbents from the liquid phase rather than filtered to avoid the potential sorption of PFAS compounds on the filtration. The centrifugation time made it impossible to study the kinetics of sorption, although previous studies of the kinetics of uptake of a wide variety of compounds onto β-CD polymers suggested uptake was rapid [17]. The centrifuged sample was sub-sampled into 2 mL autosampler vials for analysis. 

### 2.4. Sample Analysis

Liquid chromatography–quantitative time-of-flight mass spectrometry (LC-QTOF-MS) was used to perform target analysis. Duplicate analysis was performed for each sample in both negative electrospray ionization (ESI−) and positive electrospray ionization (ESI+) modes to capture anionic and neutral PFASs (ESI−) and zwitterionic PFASs (ESI+). 

Transitions were monitored for both quantification and qualification using multiple reaction monitoring mode (MRM) for each compound. The quantification of compounds was carried out by utilizing calibration standards spanning from 0.5 to 5000 ng/L (with an R-squared value greater than 0.99) through the isotope dilution method. To ensure the initial calibration of the instrument, continuous calibration checks were performed after every ten samples. All calibration standards, surrogate compounds, and mass-labeled internal standards were procured from Wellingtons Laboratories (Guelph, Ontario, CA, USA).

### 2.5. Quality Control

All adsorption experiments were performed in triplicates, with duplicate analysis conducted for each sample. Then, the average of the measurements was utilized for the subsequent adsorption capacities calculations. To assess adsorbent background contamination, adsorbent blanks were employed. Initial PFAS concentrations (C_0_) were determined through solvent blanks, which were treated the same as the adsorption samples. Method blanks were also carried out for quality control purposes. None of the three adsorbents exhibited any PFAS background contamination. C_0_ measurements obtained from the solvent blanks were employed for further analysis of adsorption and data processing.

### 2.6. Analytical Methods

The adsorption of PFASs on the tubes or other losses was assumed to be proportional to the final measured concentration and estimated using adsorbent-free blanks via the following equation:

(1) $$K_{Loss}=\frac{{C_{0}}_{-}C_{b}}{C_{b}}$$

where *C_b_* (ng·L^−1^) is the residual concentration of PFASs in blanks, and *C*_0_ (ng·L^−1^) is the expected initial concentration if no losses were observed. K_loss_ values were individually calculated for each PFAS. 

The adsorption capacity of each of the three adsorbents for every PFAS was then determined using the following equation:

(2) $$q_{e}=\frac{C_{0}\times v-(1+K_{Loss})\times C_{f}\times v}{m}$$

where *q_e_* (ng·mg^−1^) is the amount of PFAS on the solid phase at equilibrium; $C_{f}$ (ng·L^−1^) is the equilibrium (final) concentration of PFAS after exposure to the sorbent; *C*_0_ (ng·L^−1^) is the initial PFAS concentration (100 ng·L^−1^ and 1000 ng·L^−1^ here); *v* is the volume (l); and *m* is the dry mass of the adsorbent (mg). 

*q_e_* values were utilized to calculate equilibrium distribution coefficients into the adsorbents, K_d_, for each PFAS using the following equation:

(3) $$K_{d}=\frac{q_{e}\;(ng\cdot {mg}^{-1})}{C_{f}\;(ng\cdot L^{-1})}\times 1000\;(\frac{mg}{g})\;$$

where K_d_ is the adsorption coefficient (L·g^−1^), and *C_f_* (ng·L^−1^) is the measured PFAS concentration at equilibrium. The measured K_d_ values are summarized in Table S2.

## 3. Results

The adsorption affinities of three adsorbents for each PFAS were assessed by calculating K_d_ values, which were derived from adsorption experiments conducted with initial PFAS concentrations of 100 ng·L^−1^ and 1000 ng·L^−1^, as well as adsorbent concentrations of 10 mg·L^−1^. K_d_ values obtained from each initial concentration showed no statistically significant differences, thereby confirming linear adsorption isotherm behavior. Subsequently, the K_d_ values obtained from both sets of experiments were averaged and utilized to compare the adsorption affinity of each adsorbent for each PFAS. 

**Figure 2:**
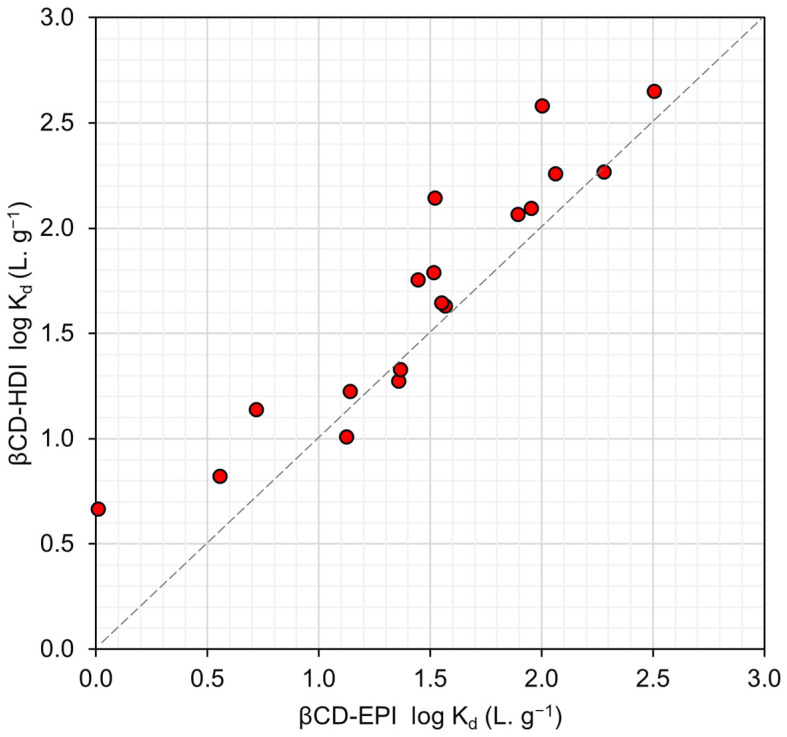
β-CD-HDI log K_d_ vs. β-CD-EPI log K_d_.

Both β-CD-HDI and β-CD-EPI exhibited moderate adsorption capabilities for various PFASs, including anionic and nonionic compounds and one zwitterionic compound. Both β-CD-HDI and β-CD-EPI exhibited removal efficiencies around or less than 10% for the majority of PFAS compounds. β-CD-EPI removed 19.2% of AmPr-FHxSA when the initial concentration was 100 ng·L^−1^. β-CD-HDI removed 12.2% of N-MeFOSAA, while the removal efficiency of β-CD-EPI for the same compound was 13.7%. Moreover, β-CD-HDI and β-CD-EPI removed 13.4% and 14.26% of PFHpA, respectively. The percentage removals of PFOS were calculated as 11% and 18.7% for β-CD-HDI and β-CD-EPI, respectively. The log K_d_ ranged from 0.6 L·g^−1^ to 2.6 L·g^−1^. These two adsorbents displayed comparable adsorption affinities for the same set of compounds. Figure 2 illustrates the correlation between the log K_d_ values of β-CD-HDI and β-CD-EPI, revealing similar K_d_ values across different PFASs in both β-CD-HDI and β-CD-EPI.

**Figure 3:**
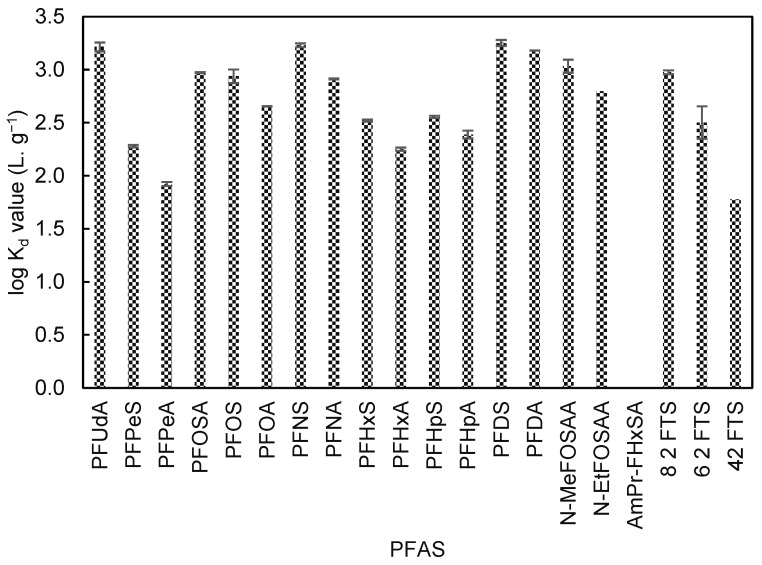
β-CD-Cl log K_d_ values (L·g^−1^) for different PFASs. No adsorption was observed for AmPr-FHxSA. Error bars represent relative error in log scale.

β-CD-Cl exhibited stronger adsorption affinities towards anionic and nonionic PFASs, compared to β-CD-HDI and β-CD-EPI. It removed 28.1% of N-MeFOSAA, while β-CD-HDI and β-CD-EPI removed 12.2% and 13.7% of the same compound. β-CD-Cl removal efficiencies for PFOS, PFHpA, and PFOA were 50.44%, 66.41%, and 75%, respectively. The log K_d_ values ranged from 1.9 L·g^−1^ to 3.3 L·g^−1^. However, no significant removal of zwitterionic compounds was observed. Figure 3 displays β-CD-Cl log K_d_ values for different PFASs.

## 4. Discussion

### 4.1. Hydrophobic Interactions

**Figure 4:**
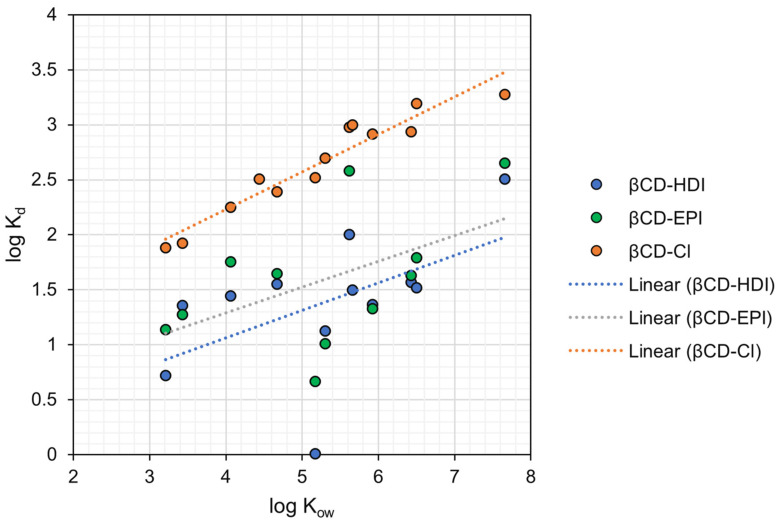
The log K_d_ vs. log K_ow_ of PFASs tested in this work. Compounds for which log K_ow_ values were unavailable were excluded from the analysis. The log K_ow_ values were sourced from the existing literature [38].

The measured adsorption revealed a positive correlation between the log K_ow_ of targeted PFASs (Table S3) and the log K_d_ values of the three adsorbents in this study. Figure 4 demonstrates the correlation between log K_d_ and the logarithm of the octanol/water partition coefficient (log K_ow_) of different PFASs. β-CD-Cl was observed to be a stronger adsorbent for most of the tested PFASs, compared to β-CD-HDI and β-CD-EPI. The strong correlation between K_ow_ and K_d_ for β-CD-Cl suggests that hydrophobic interactions are the primary mechanisms of sorption. Our finding highlights the role of hydrophobic interactions in the adsorption mechanism and are consistent with findings of prior studies regarding the effect of fluorinated carbon chain length on hydrophobic adsorption affinities [32]. β-CD is also recognized for its hydrophobic inner cavities, making it an excellent foundational component for adsorptive materials designed to capture hydrophobic organic contaminants. 

The correlations of PFAS K_d_ to K_ow_ were more scattered for β-CD-HDI and β-CD-EPI, suggesting that additional factors are likely important. These were not investigated further, due to the weaker sorption onto these sorbents. The presence of aromatic rings in β-CD-Cl further enhanced the hydrophobic nature of this adsorbent, compared to β-CD-HDI and β-CD-EPI (see Figure 1).

### 4.2. Effect of Surface Charge

**Figure 5:**
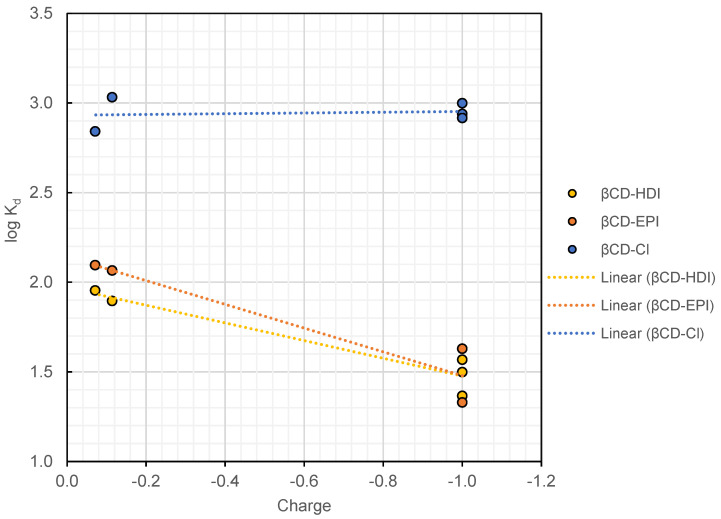
Effect of PFAS charge on log K_d_ values. The data points correspond to PFOS, PFNA, 8:2 FTS, N-MeFOSAA, and N-EtFOSAA, all of which share the same chain length of 8 fluorinated carbons but exhibit different charges. Surface charges were extracted from literature [39].

While previous studies suggested that the surface charge of both the adsorbent and the PFAS play a role in adsorption affinities [17], this effect was not observed in β-CD-Cl. Figure 5 represents log K_d_ vs. the charge of five PFAS (Table S3), PFOS, perfluorononanoic acid (PFNA), 8:2 fluorotelomer sulfonate (8:2 FTS), N-methylperfluorooctane sulfonamido acetic acid (N-MeFOSAA), and N-ethylperfluorooctane sulfonamido acetic acid (N-EtFOSAA), all of which share a chain length of eight fluorinated carbons (and exhibit similar hydrophobicity). β-CD-Cl demonstrated comparable K_d_ values for all five PFAS, regardless of their charges. This observation suggests that hydrophobic interactions play a dominant role in the adsorption mechanism of β-CD-Cl. However, some effects of charge were observed in the case of β-CD-HDI, and β-CD-EPI and these adsorbents illustrated higher K_d_ values for less negative PFASs. Meanwhile, both β-CD-HDI and β-CD-EPI exhibited a slight adsorption of AmPr-FHxSA, a zwitterionic compound carrying a positive charge at the neutral experimental pH. β-CD-HDI and β-CD-EPI log K_d_ values for AmPr-FHxSA were 1.5 and 2.1, respectively. However, β-CD-Cl did not illustrate any significant removal of this compound, suggesting that positively charged species are not adsorbed by this adsorbent. 

### 4.3. A Comparison between β-CD-Cl and Other Sorbents

**Figure 6:**
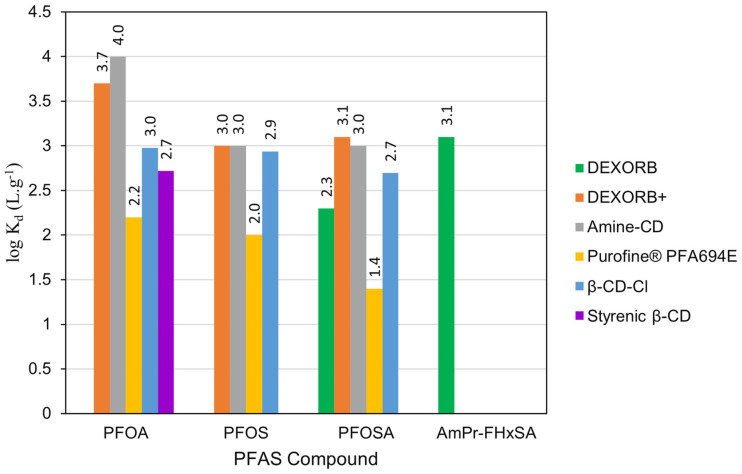
A comparison between the performance of β-CD adsorbents in this study and other adsorbents from literature [17].

To assess the performance of β-CD-Cl in comparison to other adsorbents used in prior studies, K_d_ values for four PFASs common to both this study and previous research were compared (Figure 6). β-CD-Cl outperformed DEXORB, a commercial β-CD adsorbent, in the removal of PFOA, PFOS, and perfluorooctane sulfonamide (PFOSA). Moreover, β-CD-Cl demonstrated higher PFOA removal compared to the recently explored styrenic β-CD polymer, which serves as a non-fluorinated β-CD adsorbent for PFAS removal [28]. However, DEXORB performed better for the zwitterionic compound, AmPr-FHxSA. Meanwhile, DEXORB+, amine-CD, and β-CD-Cl demonstrated comparable K_d_ values for PFOS and PFOA. This comparison highlights the varying performances of adsorbents for any specific PFAS, emphasizing the effect of PFAS chemistry and also adsorbent properties on adsorption affinities. The similar adsorptions of a number of PFASs by β-CD-Cl compared to the commercial sorbents are encouraging, particularly since the commercial sorbents typically contain fluorine, which generates some environmental concerns. 

## 5. Conclusions

In this study, we assessed the adsorption affinities of three different β-CD adsorbents for various PFASs. Key findings included the differential performances of adsorbents for specific PFASs. β-CD-HDI and β-CD-EPI demonstrated moderate adsorption of PFASs, with a better performance observed for perfluoroalkanoic acids and perfluoroalkane sulfonates. β-CD-Cl, a previously unreported potential sorbent, was also synthesized and exhibited stronger adsorption affinity towards anionic and nonionic PFASs, although it did not exhibit significant removal of zwitterionic compounds.

A positive correlation between log K_d_ and log K_ow_ of PFASs was observed, indicating the influence of hydrophobic interactions in PFAS adsorption. Both the magnitude of sorption and the correlation with K_ow_ were the strongest in β-CD-Cl. Prior research has indicated that the surface charges of both adsorbents and target compounds often play a significant role in adsorption affinities. Some charge-related effects were observed in both β-CD-HDI and β-CD-EPI, where less negatively charged PFASs exhibited higher K_d_ values. β-CD-HDI and β-CD-EPI also exhibited adsorption of AmPr-FHxSA, a zwitterionic PFAS with a positive charge at the pH of the studies. This observation suggests that the charge of PFASs may play a role in the adsorption mechanism of β-CD-HDI and β-CD-EPI. This effect was not observed in β-CD-Cl, in which comparable affinities were found for anionic PFASs with different charges.

A comparison between β-CD-Cl and other adsorbents from previous studies revealed that β-CD-Cl is a promising novel adsorbent that has adsorption affinities comparable to those of fluorinated adsorbents without the use of fluorine. This promising initial result suggests additional studies should be considered to evaluate sorption, potential separation technologies using the sorbent, and potential environmental risks of the adsorbent. 

## Data Availability

The data presented in this study are available upon request from the corresponding authors.

## Supplementary Materials

The following supporting information can be downloaded at: https://www.mdpi.com/article/10.3390/toxics12040264/s1, Figure S1: FTIR spectra of β-CD crosslinked with epichlorohydrin (EPI); Figure S2: FTIR spectra of β-CD crosslinked with hexamethylene diisocyanate (HDI); Figure S3: FTIR spectra of β-CD-Cl; Figure S4: N2 adsorption by β-CD-Cl and BET analysis; Figure S5: Scanning electron microscope images of β-CD-Cl showing particle size and structure; Figure S6: X-ray Diffraction Pattern of β-CD-Cl; Table S1: List of targeted analytes with respective mass-labeled standards analyzed in this study; Table S2: PFAS adsorption affinities on β-CD-Cl; Table S3: The log K_ow_, number of fluorinated carbons, surface charge, and log K_d_ values of PFAS in this study [39].
