# Supplementary material for: Application of β-Cyclodextrin Adsorbents in the Removal of Mixed Per- and Polyfluoroalkyl Substances
Source: Toxics. 2024 Mar 31;12(4):264. doi: 10.3390/toxics12040264 (PMC11054934; doi:10.3390/toxics12040264)
Supplement: Supplementary file 1 [file toxics-12-00264-s001.zip › toxics-2825012-supplementary.pdf]

Supplementary materials for Application of β-Cyclodextrin Adsorbents in Removal of Mixed Per- and Poly Fluoroalkyl Substances

Elham Abaie [1], Manish Kumar [1], Naveen Kumar [1], Yilang Sun[1], Jennifer Guelfo [1], Yuexiao Shen [1], and Danny Reible [1,2,*]

[1] Civil, Environmental, and Construction Engineering Department, Texas Tech University; elham.abaie@ttu.edu (E.A.); maniskum@ttu.edu (M.K.); naveen.kumar@ttu.edu (N.K.); jennifer.guelfo@ttu.edu (J.G.); Yuexiao.shen@ttu.edu (Y.S.)
[2] Chemical Engineering Department, Texas Tech University
* Correspondence: danny.reible@ttu.edu

Texts

Synthesis

β-CD-EPI synthesis: The synthesis was conducted according to the literature with minor modifications[1]. A solution of 20% sodium hydroxide in 250 ml distilled water was prepared. β-CD (100 g) and EPI (152 g) were mixed in this solution at 50°C. The resulting mixture was slowly added to magnetically stirred liquid paraffin at 40°C. After allowing it to react overnight, the formed beads were separated from the liquid paraffin. The beads were then washed successively with hexane, acetone, and distilled water. Subsequently, the β-CD-EPI was dried under vacuum at 60°C for two days. Finally, it was rinsed with methanol and distilled water, followed by another round of vacuum drying at 60°C for two days.

β-CD-HDI synthesis: Adsorbent was synthesized according to the literature with minor modifications [2]. To synthesize β-CD-HDI, 2.0 g of β-CD was mixed with one drop of dibutyltin dilaurate and 15 ml of dimethylformamide (DMF). The mixture was stirred in a nitrogen atmosphere. A solution of 1.2 g HDI in 5 ml of DMF was prepared separately. The solution was added drop by drop to the mixture of β-CD and heated at 70 ˚C for 24 hours in a nitrogen atmosphere. The content of the flask, consisting of the β-CD polymer, was poured into chloroform. The resulting suspension was stirred, and the precipitate was collected through filtration. To remove any unreacted β-CD, it was rinsed with water. The solid was then collected through filtration and vacuum dried at 60°C.

β-CD-Cl synthesis: Adsorbent was synthesized based on a previous work [3]**.** The reaction mixture was prepared by dissolving 500 mg of β-CD in 10 mL of dry DMF. The solution was then cooled to 0 °C. Subsequently, 740 mg of NaH (60%) was added to the mixture under a nitrogen atmosphere. The mixture was stirred for 15 minutes. Following this, 3.24 g of dichloroxylene was slowly added to the mixture, and the reaction mixture was warmed to room temperature. The mixture was stirred

for a duration of 5 days. The reaction was quenched by adding 5 mL of methanol, and the mixture was diluted with 100 mL of water. The resulting precipitate was filtered and washed with methylene chloride. Finally, the precipitate vacuum dried for 24 hours.

FTIR Spectroscopy Studies

β-CD-EPI: Fig S-1 presents the FTIR spectra of β-CD-EPI. Our material exhibits IR signatures consistent with those reported in the literature, specifically at 3400 cm^{-1} (O-H stretch), 3000 cm^{-1} (C-H stretch), and 1040 cm^{-1} (C-O-C stretch). These findings align with existing literature and confirm the successful synthesis of the desired product[1]. Additionally, the IR spectrum of the product closely resembles that of β-CD, indicating that the copolymer materials maintain the fundamental structural units intact.

β-CD-HDI: Figure S2 illustrates the FTIR spectra of β-CD-HDI. The peaks observed at 2920 and 2850 cm^{-1} correspond to the CH_2 stretch present in the HDI crosslinker. Furthermore, the peak at 1540 cm^{-1} indicates the formation of NHCO bonds during the polymerization process. Additionally, the appearance of a peak at 1250 cm^{-1} can be attributed to the C-O-C stretches. These results align with existing literature and provide compelling evidence for the successful synthesis of the β-CD-HDI polymer [2].

β-CD-Cl: Figure S3 illustrates the FTIR spectra of β-CD-Cl. The peak observed at 1260 cm^{-1}, is associated with C-Cl stretching from CH_2Cl. C=C stretching appeared at around 1637 cm^{-1} were attributed to the aromatic ring, while the peak at 2920 cm^{-1} belonged to stretching vibrations of C-H in the aromatic ring [3].

Figures

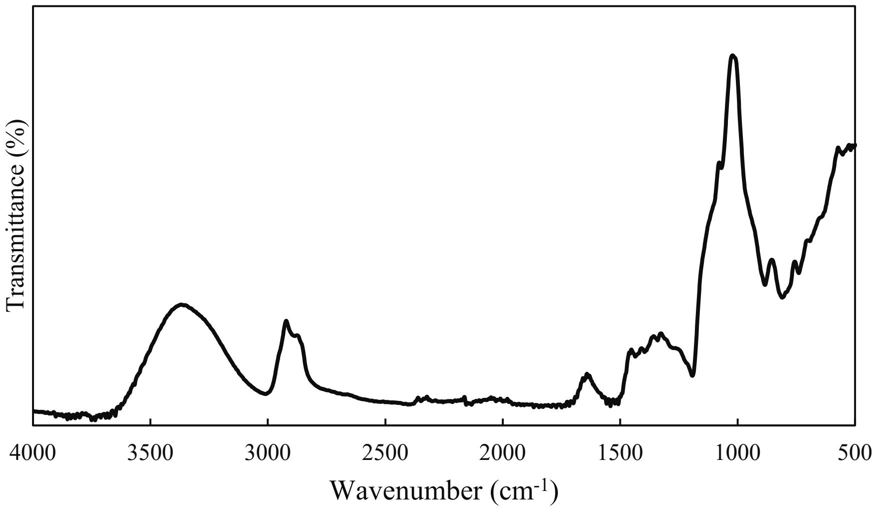

Figure S1. FTIR spectra of β-CD cross linked with epichlorohydrin (EPI)

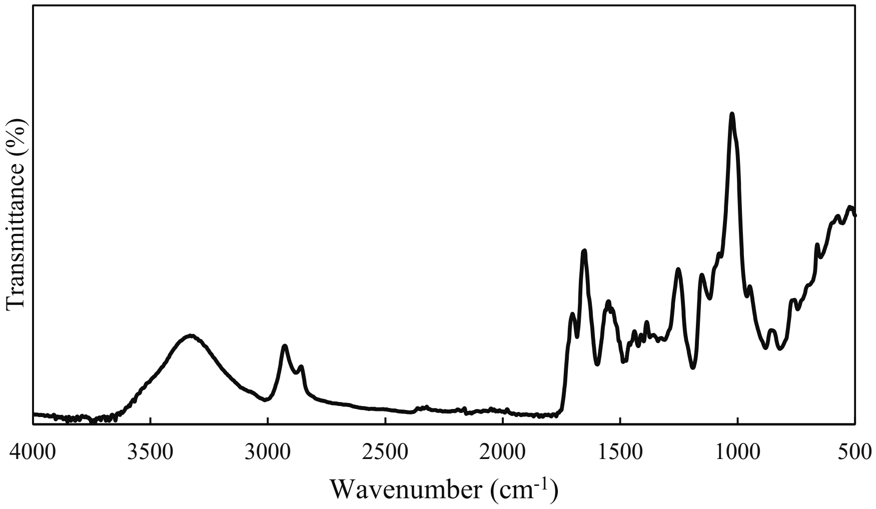

Figure S2. FTIR spectra of β-CD cross linked with hexamethylene diisocyanate (HDI)

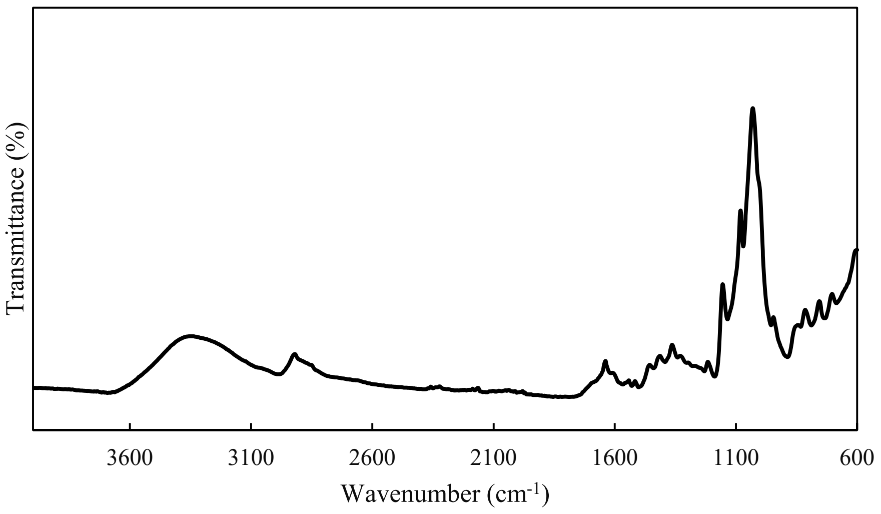

Figure S3. FTIR spectra of β-CD-Cl

Further characterization of β-CD-Cl

The β-CD-Cl was observed to be the most effective at adsorbing PFAS compounds and has not been previously characterized in the literature so was subjected to further characterization. BET analysis (Figure S4) indicated that the β-CD-Cl exhibited a modest surface area of approximately 9 m^2/gm and relatively large pores, peaking at around 20 nm but with significant pores in excess of 100 nm. As noted by Wilson et al. [4], the N_2 adsorption of β-CD polymers may not reflect the apparently much higher surface area in aqueous solutions as a result of swelling of the copolymer framework. The β-CD-Cl was dominated by particle sizes of 10-30 µm in size (Figure S5). The XRD pattern (Figure S6) reflects the amorphous structure of the β-CD-Cl.

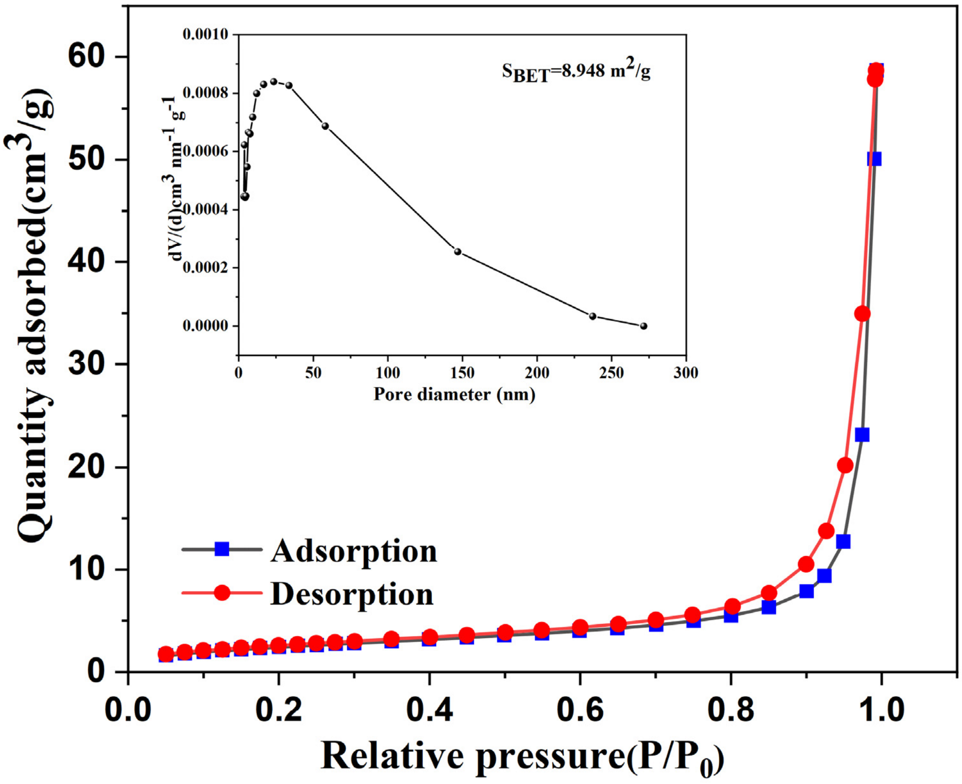

Figure S4. N_2 adsorption by β-CD-Cl and BET analysis

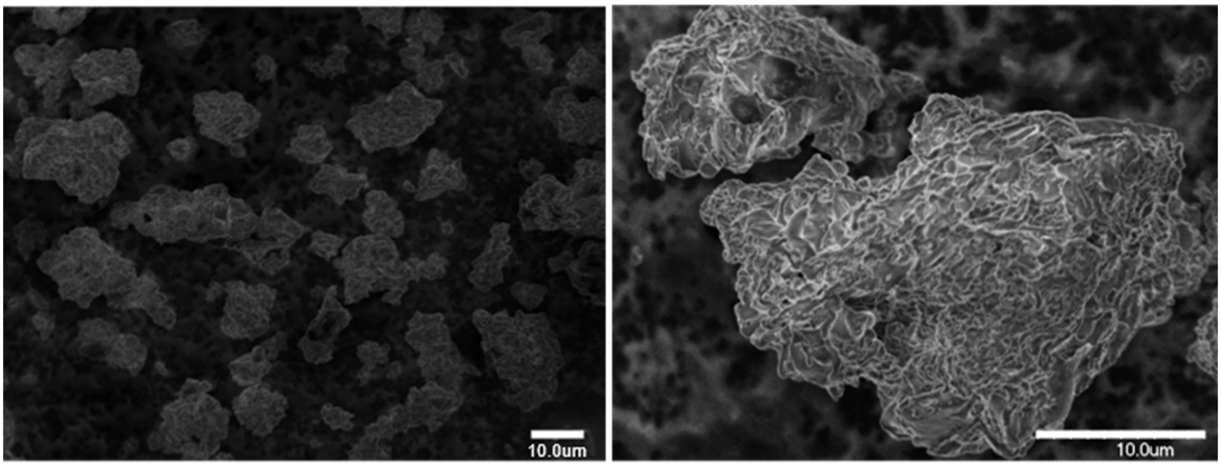

Figure S5. Scanning electron microscope images of β-CD-Cl showing particle size and structure

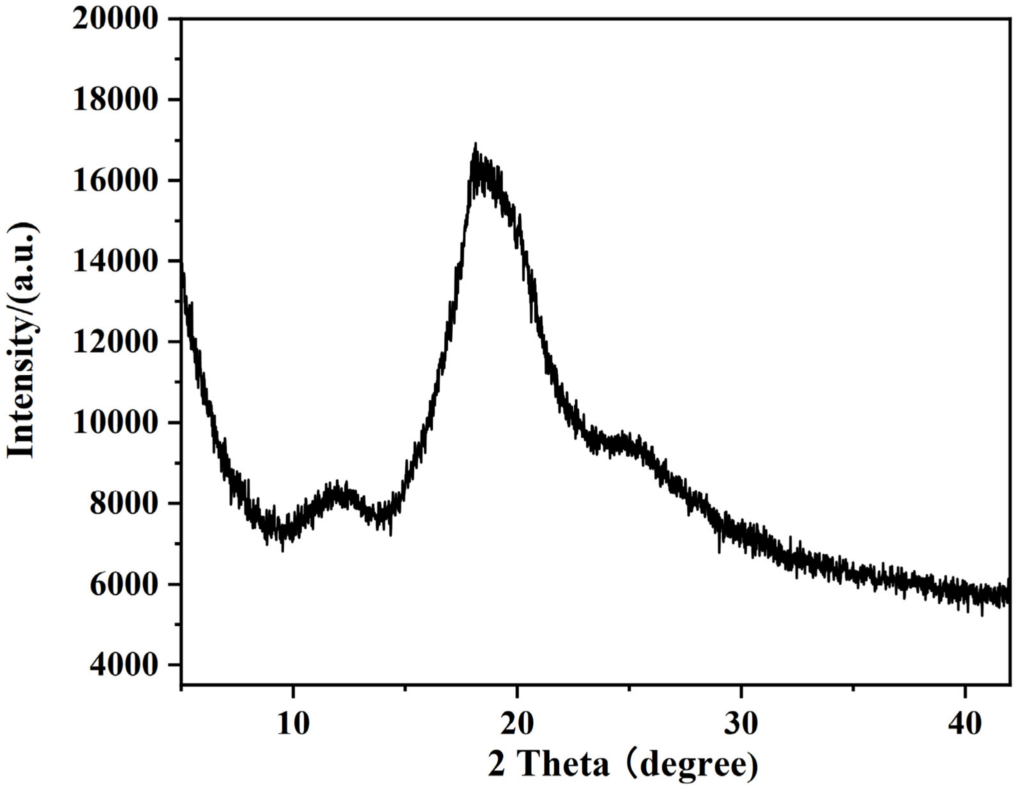

Figure S6. X-ray Diffraction Pattern of β-CD-Cl

Tables

Table S1. List of targeted analytes with respective mass-labeled standards analyzed in this study.

Analytes	Acronym	Mas Labelled Internal Standard	log K_{ow}	Retention Time(min)
**Perfluoroalkanoic acids: carboxyl functional group (COOH)**				
Perfluorobutanoic acid	PFBA	[13C3] PFBA	2.82	5.31
Perfluoropentanoic acid	PFPeA	[13C4] PFPeA	3.43	5.92
Perfluorohexanoic acid	PFHxA	[13C5] PFHxA	4.06	6.48
Perfluoroheptanoic acid	PFHpA	[13C4] PFHpA	4.67	7.03

Perfluorooctanoic acid	PFOA	[13C8] PFOA	5.3	7.49
Perfluorononanoic acid	PFNA	[13C8] PFNA	5.92	8.26
Perfluorodecanoic acid	PFDA	[13C6] PFDA	6.5	8.86
Perfluoroundecanoic acid	PFuDA	[13C7] PFUnA	7.15	9.35
Perfluorododecanoic acid	PFDoA	[13C2] PFDoA		9.95
Perfluorotridecanoic acid	PFTrDA	[13C2] PFTeDA		10.53
Perfluorotetradecanoic acid	PFTeDA	[13C2] PFTeDA		10.99
**Perfluoroalkane Sulfonates: sulfonate functional group (-SO3-)**				
Perfluorobutane sulfonate	PFBS	[13C3] PFBS	3.9	5.99
Perfluoropentane sulfonate	PFPeS	[13C3] PFBS		6.48
Perfluorohexane sulfonate	PFHxS	[13C3] PFHxS	5.17	6.85
Perfluoroheptane sulfonate	PFHpS	[13C8] PFOS		7.59
Perfluorooctane sulfonate	PFOS	[13C8] PFOS	6.3	8.19
Perfluorononane sulfonate	PFNS	[13C8] PFOS		8.69
Perfluorodecane sulfonate	PFDS	[13C8] PFOS	7.66	9.44
**Perfluoroalkane sulfonamides: Sulfonamide functional group (-SO2NH2)**				
Perfluorobutane sulfonamide	FBSA	[13C8] FOSA		6.34
Perfluorohexane sulfonamide	FHxSA	[13C8] FOSA		7.5
Perfluorooctane sulfonamide	FOSA/PFOSA	[13C8] FOSA		8.85
**Fluorotelomer Sulfonates**				
4:2 fluorotelomer sulfonate	4:2 FTS	[13C2] 4:2FTS		6.4
6:2 fluorotelomer sulfonate	6:2 FTS	[13C2] 6:2FTS		7.58
8:2 fluorotelomer sulfonate	8:2 FTS	[13C2] 8:2FTS		8.86
**Perfluoroalkane sulfonamido acetic acids**				
N-methylperfluorooctane sulfonamido acetic acid	N-MeFOSAA	[2H3] N-MeFOSAA	6.07	9.15

N-ethylperfluorooctane sulfonamido acetic acid	N-EtFOSAA	[2H5] N-EtFOSAA	6.71	9.51
**Zwitterionic PFAS**				
N-dimethyl ammonio propyl perfluorohexane sulfonamide	AmPr-FHxSA	[2 H3] NMeFOSAA		7.67
6:2 fluorotelomer sulfonamido propyl betaine	6:2 FTSA-PrB	[2 H3] NMeFOSAA		7.62
N-Trimethylammoniopropyl perfluorohexane sulfonamide	TAmPrFHxSA	[2 H3] NMeFOSAA		9.97

Table S2. PFAS compounds adsorption affinities on β-CD-Cl

Compound	log K_d (L. g^{-1})	Relative error
PFUdA	3.2	0.1
PFPeS	2.3	0.2
PFPeA	1.9	0.1
PFOSA	3.0	0.1
PFOS	2.9	0.0
PFOA	2.7	0.4
PFNS	3.2	0.0
PFNA	2.9	0.1
PFHxS	2.5	0.0
PFHxA	2.3	0.1
PFHpS	2.6	0.1
PFHpA	2.4	0.1
PFDS	3.3	0.3
PFDA	3.2	0.2
N-MeFOSAA	3.0	0.0
N-EtFOSAA	2.8	0.4
AmPr-FHxSA	---	---
8 2 FTS	3.0	0.3
6 2 FTS	2.5	0.1
42 FTS	1.8	0.6

Table S3. log K_{ow}, number of fluorinated carbons, surface charge, and log K_d values of PFAS compounds in this study. Surface charge and pKa values were obtained from literature[5].

PFAS compound	βCD-HDI	βCD-EPI	βCD-Cl	log Kow	Number of C	Surface charge	pKa
PFUdA	2.3	2.3	3.2		10	-1	
PFPeS	0.6	0.8	2.3		5	-1	0.14
PFPeA	1.4	1.3	1.9	3.43	4	-1	-0.1
PFOSA	2.0	2.6	3.0	5.62	8	-0.633	6.56
PFOS	1.6	1.6	2.9	6.43	8	-1	0.14
PFOA	1.1	1.0	2.7	5.3	7	-1	out of range
PFNS	2.1	2.3	3.2		9	-1	0.14
PFNA	1.4	1.3	2.9	5.92	8	-1	out of range
PFHxS	0.0	0.7	2.5	5.17	6	-1	0.14
PFHxA	1.4	1.8	2.3	4.06	5	-1	-0.17
PFHpS	1.1	1.2	2.6		7	-1	0.14
PFHpA	1.6	1.6	2.4	4.67	6	-1	-0.2
PFDS	2.5	2.6	3.3	7.66	10	-1	0.14
PFDA	1.5	1.8	3.2	6.5	9	-1	out of range
N-MeFOSAA	1.9	2.1	3.0		8	-0.114	3.9
N-EtFOSAA	2.0	2.1	2.8		8	-0.071	3.9
AmPr-FHxSA	1.5	2.1				0.207	6.25
8 2 FTS	1.5		3.0	5.66	8	-1	0.36
6 2 FTS			2.5	4.44	6	-1	0.36
42 FTS	0.7	1.1	1.9	3.21	4	-1	0.36

References of Supplementary Materials

[1] Shoji M, Satoshi I, Yasuyuki M, Keiko T, Kenjiro H. Adsorption and Recovery of Nonionic Surfactants by b-Cyclodextrin Polymer. Journal of Colloid and Interface Science 1996;183:118–123.

[2] Yamasaki H, Makihata Y, Fukunaga K. Efficient phenol removal of wastewater from phenolic resin plants using crosslinked cyclodextrin particles. Journal of Chemical Technology & Biotechnology 2006;81:1271–6. https://doi.org/https://doi.org/10.1002/jctb.1545.

[3] Li X, Zhou M, Jia J, Jia Q. A water-insoluble viologen-based β-cyclodextrin polymer for selective adsorption toward anionic dyes. Reactive and Functional Polymers 2018;126:20–6. https://doi.org/10.1016/j.reactfunctpolym.2018.03.004.

[4] Wilson LD, Mohamed MH, Headley J V. Surface area and pore structure properties of urethane-based copolymers containing β-cyclodextrin. Journal of Colloid and Interface Science 2011;357:215–22. https://doi.org/10.1016/j.jcis.2011.01.081.

[5] Wang C, Yan B, Munoz G, Sauvé S, Liu J. Modified clays reduce leaching of per- and polyfluoroalkyl substances from AFFF-contaminated soils. AWWA Water Science 2021;3:e1241. https://doi.org/https://doi.org/10.1002/aws2.1241.
